# *Helicobacter pylori* in Inflammatory Bowel Diseases: Active Protagonist or Innocent Bystander?

**DOI:** 10.3390/antibiotics13030267

**Published:** 2024-03-17

**Authors:** Elisabetta Bretto, Simone Frara, Angelo Armandi, Gian Paolo Caviglia, Giorgio Maria Saracco, Elisabetta Bugianesi, Demis Pitoni, Davide Giuseppe Ribaldone

**Affiliations:** 1 Department of Medical Sciences, University of Turin, 10126 Turin, Italy; elisabettaalessandra.bretto@unito.it (E.B.); simone.frara@unito.it (S.F.); angelo.armandi@unito.it (A.A.); gianpaolo.caviglia@unito.it (G.P.C.); giorgiomaria.saracco@unito.it (G.M.S.); elisabetta.bugianesi@unito.it (E.B.); davidegiuseppe.ribaldone@unito.it (D.G.R.); 2 Unit of Gastroenterology, Città della Salute e della Scienza di Torino-Molinette Hospital, 10126 Turin, Italy

**Keywords:** *H. pylori*, inflammatory bowel disease, Crohn’s disease, ulcerative colitis, immunological modulation, IBD, diagnosis, molecular biology, eradication

## Abstract

*Helicobacter pylori* (*H. pylori*) infection is a prominent entity within human infectious diseases which cause chronic gastritis, peptic ulcers, gastric malignancies, and extragastric disorders. Its persistent colonization can lead to a systemic inflammatory cascade, potentially instigating autoimmune responses and contributing to the pathogenesis of autoimmune diseases. While the specific etiopathogenesis of inflammatory bowel diseases (IBDs) is still unknown, it is widely recognized that immunological, genetic, and environmental factors are implicated. Various bacterial and viral pathogens have been implicated in the pathogenesis of IBDs. Numerous studies suggest a correlation between *H. pylori* infection and IBDs. While subject to debate, this link suggests that the bacterium’s presence somehow impacts the progression of IBDs by modifying the diversity of the gut microbiota, consequently altering local chemical profiles and disrupting the pattern of gut immune response. However, epidemiological evidence indicates a protective role of *H. pylori* infection against the onset of autoimmune diseases. Additionally, laboratory findings demonstrate *H. pylori*’s capacity to promote immune tolerance and restrict inflammatory reactions. The aim of this review is to elucidate the proposed mechanisms and confounding factors that underlie the potential association between *H. pylori* infection and IBDs.

## 1. Introduction

*Helicobacter pylori* (*H. pylori*), a widely prevalent gastrointestinal pathogen estimated to affect at least 4.4 billion individuals globally, demonstrates varying infection rates in industrialized and developing nations [1]. In industrialized regions, there is a decline in *H. pylori* infection rates, in contrast to the increasing prevalence of inflammatory bowel diseases (IBDs) [2,3].

IBDs are chronic and relapsing inflammatory conditions, including Crohn’s disease (CD) and ulcerative colitis (UC) [4]. These conditions are characterized by persistent inflammation leading to complications such as hospitalization, surgery, colorectal cancer, and disability, thereby significantly impacting the affected individuals’ quality of life [5].

Numerous epidemiological studies have consistently observed an inverse relationship between *H. pylori* infection and IBDs, even after adjusting for factors like age, ethnicity, detection methods, and prior drug exposures [2,3]. The exact mechanism of this inverse association remains uncertain, with some studies suggesting a potential protective role of *H. pylori* in IBDs by inducing systemic immune tolerance and suppressing inflammatory responses [2,3,6,7]. Moreover, specific studies have shown an association between *H. pylori* infection and the severity of IBDs in both pediatric and adult populations [3,8].

While several studies propose a potential negative correlation, conflicting opinions persist in the literature [9]. The absence of comprehensive, large-scale systematic reviews in the field poses a challenge to resolving this issue definitively. Consequently, the determination of whether *H. pylori* infection is conclusively associated with IBDs remains unresolved. The objective of this review is to investigate and shed light on this association.

A comprehensive literature search was performed by authors to identify potentially relevant articles published in English describing the relationship between *H. pylori* infection and IBDs in the PubMed, Google Scholar, and the ClinicalTrials.gov portal databases in the last ten years. The following search terms were used alone or matched with ‘AND’ or ‘OR:’ ‘*Helicobacter pylori*’, ‘Crohn’s disease’, ‘ulcerative colitis’, ‘association’, ‘role’, ‘immunomodulation’, and ‘protective factor’. Case reports and case series were excluded. Moreover, a hand-search of original articles and abstracts from recent major meetings investigating this subject was performed to review the latest results of ongoing clinical trials.

## 2. Epidemiological Insights on *H. pylori* and IBDs

The industrialization-driven environmental change is believed to play a significant role in the global rise of IBD cases. Remarkably, regions such as the Asia–Pacific, where IBD incidence has surged the most, demonstrate an inverse relationship with *H. pylori* infection rates [2,3].

Although *H. pylori* infection remains the most widespread chronic bacterial infection globally, its frequency has markedly decreased in recent decades [2,6]. This decline is largely attributed to advancements in hygiene practices and the use of targeted antibiotic therapies, which have shown considerable effectiveness in eradicating the bacterium despite the emergence of resistance [6]. Consequently, there is now a higher prevalence of *H. pylori* infection in developing countries compared to industrialized ones [2]. Conversely, IBDs are more prevalent in developed nations, likely due to the rapid industrialization and subsequent increased exposure to Western-type lifestyle [10]. This industrialization-driven environmental change is believed to play a significant role in the global rise of IBD cases [11].

## 3. Association between *H. pylori* and IBDs

Numerous studies have investigated the relationship between *H. pylori* infection and IBDs, with epidemiological research providing evidence for a reverse correlation between the declining prevalence of *H. pylori* infection in the general population and the rising occurrence of immune-mediated conditions, including IBDs and notably CD [3,6]. This highlights the potential of specific strains of *H. pylori* to contain elements that may influence the clinical course of immunologically mediated diseases, such as IBDs, through an immunomodulatory mechanism by triggering both specific and nonspecific immune responses in the human intestine [2,3,7].

## 4. Inverse Association between *H. pylori* Infection and IBDs

Over time, there has been significant documentation of the notable inverse correlation in prevalence between *H. pylori* gastric infection and IBDs, independent of age, ethnicity, and methods used for *H. pylori* detection [2,3,6].

The most recent and comprehensive meta-analysis to investigate this negative association was conducted by Castano-Rodriguez et al. analyzing 40 studies [3]. The study’s total sample comprised 6130 IBD patients and 74,659 non-IBD controls [3]. The overall analysis, covering all IBD patients (including those with CD, UC, and unclassified IBDs, IBD-U), revealed a negative association, indicating a 57% lower likelihood of IBDs (OR: 0.43, 95% CI 0.36 to 0.50, *p* < 0.0000000001) [2,3,6]. Stratified analyses by IBD subtypes confirmed this association: for CD with 2938 patients and 74,012 controls (OR: 0.38; 95% CI 0.31 to 0.47; *p* value < 0.0000000001), for UC with 2520 patients and 73,087 controls (OR: 0.53; 95% CI 0.44 to 0.65; *p* < 0.0000000001), and for IBD-U with 286 patients and 70,247 controls (OR: 0.43; 95% CI 0.23 to 0.80; *p* = 0.008) [2,3,6]. This negative association was more prominent for CD and IBD-U compared to UC [2,3,6]. Additional meta-analyses, including one focusing solely on Asian studies, corroborated these findings, demonstrating a significant inverse association between *H. pylori* infection and IBDs, with a stronger effect observed for CD compared to UC [2,6].

Age-stratified analyses confirmed a negative correlation both in adults (age > 16 years, 5459 IBD patients and 68,781 non-IBD controls; OR: 0.45; 95% CI 0.38 to 0.53; *p* < 0.0000000001) and in children (age < 16 years, 671 IBD patients and 5878 non-IBD controls; OR: 0.24; 95% CI 0.14 to 0.43; *p* < 0.0000000001), with a more apparent protective effect observed in the pediatric population [2,3,6]. An in-depth investigation into the association between *H. pylori* infection and the diagnosis of IBD in children was conducted by Kong et al. [8]. They analyzed six studies encompassing 2236 cases, revealing that, compared to the non-IBD control group (239 out of 1880, 12.7%), *H. pylori* infection was not significantly associated with IBD in children (35 out of 356, 9.8%), indicating no evidence of the bacterium’s role in the onset of IBD in pediatric patients (OR = 0.62; 95% CI: 0.34–1.12; *p* = 0.12) [8].

Finally, a robust negative correlation was identified through stratification by ethnicity, examining both the Eastern population (1304 IBD patients and 1788 non-IBD controls; OR: 0.35; 95% CI 0.26 to 0.48; *p* < 0.0000000001) and the Western population (4826 IBD patients and 72,871 non-IBD controls; P-OR: 0.46; 95% CI 0.38 to 0.55; *p* < 0.0000000001), with a more pronounced negative association observed in the former [2,3,6].

## 5. Variability in *H. pylori* Strains and IBD Risk

The risk of IBDs varies across different strains of *H. pylori* [2,7]. A meta-analysis revealed that exposure to *H. pylori* cytotoxin-associated antigen A (CagA)-positive strains was significantly linked to a reduced risk of IBDs (pOR 0.31, 95% CI 0.21–0.44), particularly for CD (pOR 0.23, 95% CI 0.15–0.35) compared to UC (pOR 0.66, 95% CI 0.34–1.27), where statistical significance was not reached [7]. Conversely, there was no notable distinction in the likelihood of IBDs between individuals exposed to *H. pylori* CagA-negative strains and those unexposed [7].

CagA is a gene located within the Cag pathogenicity island of the *H. pylori* genome, encoding the oncogenic protein CagA [12]. Despite *H. pylori* CagA-positive strains demonstrating higher pathogenic and carcinogenic potential (being found in approximately 90% of biopsies from patients with severe gastric diseases, such as peptic ulcer, mucosa-associated lymphoid tissue lymphoma (MALToma), and gastric adenocarcinoma), they have also been strongly associated with a protective role against IBDs [2,7]. Historically, *H. pylori* strains isolated from patients in the Asia–Pacific region (and more broadly in developing countries) were predominantly CagA-positive, while in industrialized nations, there was a more balanced distribution between CagA-positive and negative strains [3]. The documented decrease in exposure to CagA-positive strains in the Asia–Pacific region, particularly during childhood, may contribute to the significant rise in IBD incidence rates in these areas compared to other developed regions [2,7].

## 6. Pathogenetic Mechanisms Underlying the Protective Role of *H. pylori* against IBDs

There are several hypotheses regarding how *H. pylori* may influence the host immune response and thus alter the clinical course of IBDs [10,13].

The pathogenesis of inflammatory bowel diseases (IBDs) is complex, involving a combination of genetic, environmental, and immunological factors [3,6]. In particular, quantitative and qualitative alteration of gut microbiota could play a role in the development of IBDs. It should be emphasized that, to date, the existence of a “normal” state of gut microbiota or any specific and pathognomonic alterations for IBDs are not known. Likewise, the molecular mechanisms by which the microbiota could specifically induce alterations on the gut wall favoring the inflammatory process remain unclear [14,15]. Differentiation of naive CD4+ T cells into various T helper cell subsets, including Th1, Th2, Th17, and regulatory T (Treg) cells, plays a significant role in the inflammatory process of IBD [16]. Th17 cells, characterized by increased expression of IL-17, IL-21, IL-22, and IL-23, dominate the gut environment in IBD patients [17]. Dysregulated TGF-β signaling, often due to elevated Smad7 expression, further exacerbates intestinal inflammation by impairing Treg-mediated suppression of immune responses [18,19]. Additionally, Th9 cells contribute to inflammation by enhancing epithelial permeability, thus exacerbating the pathological processes associated with IBDs [20,21].

The available literature data suggest that *H. pylori*, along with specific helminths, could plausibly exert a protective effect against the onset or exacerbation of IBDs [2].

In this context, Maizels et al. examined how helminths are highly capable of modulating the host immune response by regulating adaptive responses, counteracting effector cell responses and facilitating healing and tissue remodeling [22].

This protective association, particularly notable in *H. pylori* strains positive for CagA, may stem from the bacterium itself or its byproducts exerting an immunomodulatory effect by eliciting both specific and nonspecific immune responses in the human intestine. This effect helps mitigate dysbiosis and inflammation by balancing the homeostasis of pro- and anti-inflammatory microorganisms, especially in individuals genetically predisposed to autoimmune responses in the intestine and other areas outside the intestine (e.g., asthma, allergic rhinitis, and allergies) [2,3,10].

Specifically, *H. pylori*, known for inducing acute and/or chronic inflammation in the gastric mucosa, may induce the systemic release of cytokines that downregulate the immune response, thereby modulating autoimmune processes [10]. Elevated levels of various cytokines, including IFN-γ, TNF, IL-1β, IL-6, IL-7, IL-8, IL-10, and IL-18, have been observed in the gastric epithelial cells of *H. pylori*-infected individuals compared to uninfected ones [23].

**Figure 1:**
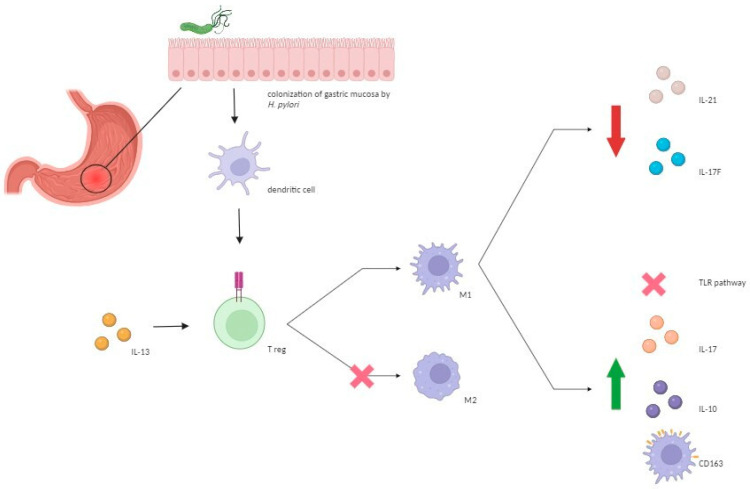
Molecular mechanisms underlying the protective role of *H. pylori* against IBDs. *H. pylori* induces the modulation of T helper 17/Treg immunological response by inducing a shift from M1 to M2 macrophage lineage which reduces levels of interleukin IL-17F and IL-21, suppresses TLR mediated signaling pathways, and increases the expression of IL-13, IL-10, and CD163, leading to anti-inflammatory effects. *H. pylori*: *Helicobacter pylori*; IL: interleukin; TLR: Toll-like receptor; Treg: regulatory T; M: macrophage.

Upon the activation of Toll-like receptors (TLRs) by *H. pylori*, dendritic cells (DCs)—crucial in modulating adaptive immunity—can activate T cells, potentially inducing either a Th1 or Th2/Treg cell response by generating IL-12 or IL-10, respectively [24]. As we already mentioned, the importance of Treg in the pathogenesis of IBDs is widely recognized [25,26]. Thus, a protective role of *H. pylori* infection against IBDs may be due to the ability of this microbe to downregulate pro-inflammatory immune pathways such as Th1/Th17 pathway by inducing a shift from M1 to M2 macrophage lineage [13,27,28]. This shift is associated with decreased levels of IL-17F and IL-21, suppression of TLR-mediated signaling pathways, along with increased expression of IL-10 and Treg cells, possibly due to elevated plasma IL-13 levels [27,28]. Moreover, increased anti-inflammatory responses may occur through elevated expression of CD163, a scavenger receptor primarily found on macrophages and monocytes, which is positively regulated by IL-10 and is upregulated during the M2/M1 phenotype switch, indicating its involvement in inflammatory processes [29,30]. Studies showed that individuals infected with *H. pylori* have increased numbers of CD163+ and CD163+/IL-10+ M2 monocytes, with higher IL-10 production by M2 cells (Figure 1) [31]. Regarding the CagA-positive strain, it has been observed to elicit a higher production of beta-defensins and other antimicrobial substances compared to CagA-negative strains [7,32]. This observation is particularly intriguing given the documented alteration in defensin production seen in IBDs, particularly in the pathogenesis of CD [7,33,34].

**Figure 2:**
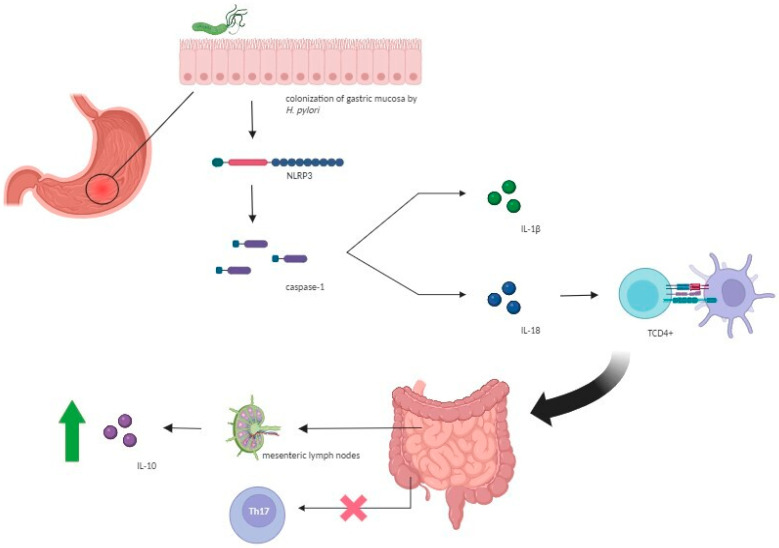
Role of the NLRP3 inflammasome in protection against inflammatory bowel diseases. Pathogen-associated molecular patterns from *H. pylori* trigger NLRP3 inflammasome activation, which upregulates caspase-1 expression, thereby leading to the activation of interleukin IL-18 and IL-1β cytokines. IL-1β as a potent pro-inflammatory agent and IL-18 by controlling responses mediated by TCD4+ cells promote extragastric immunomodulation by suppressing the Th-17 response in the cecum and increasing IL-10 in the mesenteric lymph nodes. *H. pylori: Helicobacter pylori*; IL: interleukin; NLRP3: NLR family pyrin domain containing 3.

Additionally, pathogen-associated molecular patterns derived from *H. pylori* trigger the NLR family pyrin domain containing 3 (NLRP3) inflammasome, resulting in responses linked to anti-inflammatory effects [13]. NLRP3 inflammasome is a multiprotein complex that plays a crucial role in the innate immune response [13]. Upon activation, NLRP3 upregulates caspase-1 expression, leading to the activation of interleukin IL-18 and IL-1β cytokines [24,35,36]. While IL-1β presents itself as a potent pro-inflammatory agent, IL-18 has regulatory properties and controls responses mediated by TCD4+ cells [13]. This upregulation of cytokines promotes extragastric immunomodulation by suppressing the T helper 17 subpopulation and increasing Treg cells, transforming growth factor-β, and mucins [13]. Studies with animal models have shown that NLRP3 activation induced by *H. pylori* infection appears to improve the prognosis of IBDs [37]. In vitro studies investigating the effect of *H. pylori* colonization in mice against experimental colitis observed that mice exposed to the bacterium developed less severe forms of dextran sulfate sodium (DSS)-induced colitis, with significantly milder inflammation and epithelial changes [37]. These results could be attributed to an upregulation of IL-10 in the mesenteric lymph nodes and suppression of the Th-17 response in the cecum of the infected mice, illustrating an extragastric immune-modulatory effect of the bacterium, an immunological crosstalk between the upper and lower gastrointestinal tract, and providing mechanistic support for the epidemiological observation of a negative association between *H. pylori* status and the risk of IBDs (Figure 2) [38].

Finally, Kayali et al. suggested that *H. pylori* may serve as an indicator of increased susceptibility to other gastrointestinal infections or bacteria, particularly during childhood [10]. They propose that this protective immunomodulatory effect is likely attributable the combined presence of these pathogens, rather than *H. pylori* alone. This potential of infectious agents to provide protection against autoimmune disorders is known as the “hygiene hypothesis” [2,39]. According to this concept, limited exposure to antigenic stimuli from infectious agents, including *H. pylori*, during infancy may reduce microbiome diversity, thus altering the T helper immune response orientation [2,39]. In this context, *H. pylori*, along with other gastrointestinal pathogens, could promote greater microbiome diversity and bolster intestinal barrier integrity [2].

## 7. Controversial Opinions on the Role of *H. pylori* in IBDs: Protective or Provocateur Agent?

While numerous studies document the potential protective role of *H. pylori* against IBDs, others highlight its active involvement in the clinical course of the disease, particularly in CD [9,40,41].

According to these findings, *H. pylori* can exert harmful effects on the ileal and colonic mucosa through the production of cytotoxins and urease [9,40]. Moreover, gastric infection can lead to an autoimmune-like reaction in the stomach with the production of anti-Lewis X and/or Y antibodies, which may possess systemic autoreactive properties, thereby altering the natural history of the disease [9,40]. An additional mechanism could be the induction of platelet activation and aggregation, which may result in microthrombi formation in gastric venules as well as in the colon, thus leading to ischemic damage and ulcer formation [9,40]. Furthermore, *H. pylori* infection can activate MALT, shifting the immune response from local to generalized, thus promoting or sustaining colonic inflammation [9,40]. It has been demonstrated that specific T cell clones for *H. pylori* are increased in the intestinal mucosa during active disease in IBD patients compared to peripheral venous blood (*p* = 0.0039) or the intestine in the absence of inflammation [42]. This pathogenetic mechanism may also be triggered by *H. pylori* antigens promoting intestinal activation through fecal passage [9,38].

## 8. *H. pylori* and IBD Course

The potential role of *H. pylori* infection in CD’s clinical course has been explored by Püspök et al., revealing a potential association between *H. pylori* infection and a higher frequency of surgical interventions and fewer exacerbations in non-smokers [9,40]. Conversely, patients without *H. pylori* infection experienced more frequent disease exacerbations but less frequently required surgical intervention [9]. These findings suggest that the decreased clinical activity observed in IBD patients with *H. pylori* infection may be linked to an elevated rate of resections, resulting in prolonged postoperative remissions and reduced exacerbations [9]. However, an analysis adjusting for the number of intestinal resections failed to demonstrate significant differences in the number of exacerbations per year between IBD patients with and without *H. pylori* infection [9].

In the same cohort of 131 IBD patients, those with *H. pylori* positivity exhibited a higher incidence (*p* = 0.03) of small bowel involvement compared to *H. pylori*-negative individuals. Nonetheless, statistical significance was not maintained after correction for multiple testing [9]. Similarly, the group of IBD patients with *H. pylori* infection appeared to have a higher incidence of perianal disease compared to those without *H. pylori* infection; however, statistical significance was not achieved after adjusting for disease duration (19 [38%] vs. 16 [19.8%]; *p* = 0.23) [9].

## 9. Potential Implications of *H. pylori* Eradication on the Course of IBDs

The existing data suggest a potential link between *H. pylori* eradication and the onset of CD, possibly mediated by changes in the Th1 and Th2 immune responses [39,40]. As we already mentioned, chronic *H. pylori* infection may skew the immune response towards a Th2 pattern; however, eradication could shift it towards a Th1 pattern, by decreasing Th2 cytokines (such as IL-4, IL-5, IL-6) and increasing pro-inflammatory Th1 cytokines, thereby triggering the onset of CD in genetically susceptible individuals [39].

A population study conducted on approximately 80,000 patients in Taiwan demonstrated that patients with peptic ulcer undergoing *H. pylori* eradication therapy were at a higher risk of developing IBDs, particularly CD, and/or other autoimmune diseases (OR 2.36; 95% CI 2.14–2.59) compared to those who did not receive any eradication therapy (OR 1.91; 95% CI 1.73–2.11) [2].

Although these data suggest a potential protective role against the onset of CD in genetically predisposed individuals, it is important to acknowledge that *H. pylori* has been classified by the WHO as a class I carcinogen, indicating it is a confirmed human carcinogen [10]. Consequently, when *H. pylori* positivity is detected, antibiotic eradication therapy is always recommended, even in patients with IBDs [43].

Two case reports suggest a 3–6-month interval between *H. pylori* eradication and CD onset [43].

Precautions for CD-prone individuals undergoing *H. pylori* eradication include fecal calprotectin monitoring and regular follow-up to monitor the onset of CD relapse symptoms [39].

## 10. Effect of Common Medical Treatments Used in IBDs on *H. pylori* Infection

The effect of traditional drugs used in IBD on *H. pylori* infection was addressed by Castano-Rodriguez et al. in the aforementioned meta-analysis through a stratified analysis, considering their usage in patients with IBD of various pharmacological classes and comparing them with non-IBD controls and IBD patients who were not administered drugs [3]. Regarding mesalazine (557 patients; OR: 0.51; 95% CI 0.33 to 0.80; *p* = 0.003) and sulfasalazine (702 patients; OR: 0.41; 95% CI 0.26 to 0.65; *p* < 0.0000000001), the results obtained demonstrated that treatment with such drugs may lead to spontaneous eradication of *H. pylori* [3]. The efficacy of these drugs in “preventing” *H. pylori* infection remains a subject of debate due to conflicting findings [3,44]. While some studies argue that sulfasalazine, as opposed to 5-aminosalicylic acid (5-ASA), could lower the occurrence of *H. pylori* infection, others hold a different view [3,45]. The precise mechanisms by which these medications prevent *H. pylori* infection are not completely understood, but they may include direct interference with germ adhesion to the gastric mucosa or exerting immunomodulatory effects [3]. Regarding corticosteroids (268 patients; OR: 0.30; 95% CI 0.22 to 0.42; *p* value < 0.0000000001) and antibiotics—in particular metronidazole—(117 patients; OR: 0.22; 95% CI 0.12–0.40; *p* < 0.0000000001), the negative association between *H. pylori* infection and IBDs appeared significantly increased [3,46]. These data tend to eliminate a potential bias, demonstrating how the inverse prevalence association is confirmed even in populations of non-naive IBD patients already treated with antibiotics or immunomodulatory drugs [3]. There are no available data on the effect of using biotechnological drugs in a large population of IBD patients on *H. pylori* infection.

## 11. *H. pylori*, IBDs, and Colorectal Cancer

Patients with IBDs have a well-established elevated risk of colorectal cancer compared to the general population [47].

Moreover, numerous studies have explored the potential involvement of *H. pylori* infection in the oncogenic pathway leading to colorectal cancer [40,48,49]. A meta-analysis of 13 studies was conducted by Zhao et al., indicating that *H. pylori* infection may elevate the risk of colorectal cancer in individuals without IBDs (OR 1.49; 95% CI 1.17–1.91) [40,48]. Supporting this notion, Kapetanakis et al. demonstrated the presence of *H. pylori* in colon neoplastic tissue in 82.9% of non-IBD patients (34 out of 41) with colorectal cancer [40,50]. Moreover, Sonnenberg et al. found that compared to normal gastric mucosa, *H. pylori* gastritis is more prevalent among patients with hyperplastic polyps (OR = 1.24; 95% CI: 1.18–1.30), adenomatous polyps (OR = 1.52; 95% CI: 1.46–1.57), advanced adenomas (OR = 1.80; 95% CI: 1.69–1.92), villous adenomas, or adenomas with high-grade dysplasia (OR = 1.97; 95% CI: 1.82–2.14), and adenocarcinomas (OR = 2.35; 95% CI: 1.98–2.80) [40,49].

This association is underpinned by mechanisms involving enhanced cell proliferation and altered apoptotic processes within neoplastic tissue, distinct from normal colonic mucosa [40,48]. At a molecular level, *H. pylori*-induced gastrin release can enhance the production of ammonia, pro-inflammatory cytokines, and growth factors, such as TGF-α and EGF, COX-2 overexpression, and PI3-kinase-mediated tyrosine phosphorylation of E-cadherin and β-catenin, across various gastrointestinal tract sites, including the colon [40,48]. The interplay between cytokines, growth factors, and COX2 contributes to heightened prostaglandin E2 release by inflamed or neoplastic tissue, thereby promoting tumor progression through mechanisms such as increased cell proliferation, angiogenesis, diminished apoptosis, and enhanced mutagenesis [48]. Additionally, *H. pylori* infection also leads to the recruitment of bone mar-row-derived stem cells (CD34+), which ultimately facilitates colon cancer progression [40].

Consequently, it has been suggested that eradicating *H. pylori* could potentially inhibit IBD-related or non-IBD-related colon neoplasia as well as obviously protect against gastric cancer, given that, as previously mentioned, this bacterium represents a significant risk factor for the development of such a leading cause of cancer-related mortality worldwide [40].

## 12. Conclusions

The complex relationship between *H. pylori* infection and IBDs is a subject of ongoing research and debate in the scientific community.

Our systematic review of the literature has uncovered numerous studies evaluating the correlation between *H. pylori* infection and IBDs. Most of these studies indicate a reduced incidence of *H. pylori* infection in patients with IBDs compared to control groups, suggesting a potential protective role of the bacterium against the development of immune-mediated diseases, particularly when considering the CagA-positive strain, regardless of age, ethnicity, previous treatment with corticosteroids, antibiotics and mesalazine [2,3,6,7].

Several hypotheses have been proposed to elucidate how *H. pylori* may confer protection against T cell-related immune-mediated diseases like IBDs [6,7,13,40]. Immunomodulation by *H. pylori*, particularly strains expressing the CagA virulence factor, appears pivotal in eliciting Treg cell responses and attenuating pro-inflammatory pathways implicated in IBD pathogenesis [7]. Additionally, studies using murine models suggest that *H. pylori* colonization can mitigate experimental colitis, further supporting its immunoregulatory role [37]. These associations are extensively documented in the studies cited in this review [13,24,27,28]. It is interesting that some mediators of inflammation are effective targets of advanced therapies for severe forms of IBDs that are currently available or still under investigation [13].

Despite evidence suggesting a protective role for *H. pylori*, conflicting findings highlight potential risks, particularly in CD [9,41]. Some studies underscore the possible existence of pathogenetic mechanisms through which *H. pylori* infection may, via systemic inflammatory and autoimmune mechanisms, perpetuate intestinal inflammation [9,40].

Studies supporting the theory that *H. pylori* is an active agent in IBDs are few, dated, and require further updating, even in view of the enormous strides made in the diagnostic and therapeutic pathway of IBDs. Testing for the presence of *H. pylori* during new diagnosis of IBDs, or verifying its presence and eradication in the patient’s history, could be an effective option to assess the relationship between the bacterium and IBDs by stratifying the subtypes of IBD, disease location, incidence of flare-ups and surgery, development of extra intestinal manifestations, and responses to advanced therapies.

It has been hypothesized how two precautions can be taken in patients at higher risk of developing CD (such as those with a family history of IBDs) in anticipation of *H. pylori* eradication:

- Fecal calprotectin prior to initiating eradication treatment, following the onset of any gastrointestinal disorder and by default at 3–6 months after eradication (this is the time interval by which CD occurred after *H. pylori* eradication in the two cases reported in the literature) should be monitored. Fecal calprotectin is believed to be a reliable biomarker, even in these patients, for early screening of CD because it is not found to be significantly increased during chronic *H. pylori* infection.
- Patients, educated about CD symptoms, should be followed-up once a month for the first six months after eradication [39].

The study of interactions between biotechnological drugs and IBDs could be another important field of research, particularly investigating the interaction between this category of drugs and any effects on their efficacy associated with the eradication of *H. pylori*.

In summary, caution is advised in interpreting the findings of our review due to several limitations present in the literature data. Firstly, the majority of included articles were conducted in Europe and Asia, with insufficient data from other regions and developing countries. Furthermore, many studies did not report the participants’ prior history of *H. pylori* treatment, potentially leading to an underestimation of *H. pylori* infection rates if participants had been previously treated. Additionally, heterogeneity among studies in terms of IBDs and *H. pylori* diagnosis methods may introduce limitations to the reliability of the findings. It is also important to recognize the differing inflammatory mechanisms between the two subgroups of IBD, with CD characterized by a Th1-driven immune response and UC associated with a Th2-driven response. Moreover, limited data are available on the prevalence of virulent *H. pylori* strains in IBD patients. While some studies suggest that most *H. pylori* seropositive CD patients are infected with CagA-positive strains, further investigation is warranted as these strains may elicit intense host responses, potentially altering immune-regulatory lymphocyte activity. Therefore, more randomized controlled studies are needed to enhance the reliability of the results, considering various influencing factors such as age, sex, population, ethnic, and regional differences, genetic predisposition to IBD, IBD subtypes (UC or CD) and disease activity, the type of *H. pylori* detection test, virulence of *H. pylori* strains (CagA-positive or CagA-negative), and *H. pylori* eradication outcomes (treatment efficacy and adverse events).

We hypothesize that *H. pylori* infection could indicate an increased likelihood of exposure to other gastrointestinal infections or bacteria, collectively exerting an immunomodulatory effect. Nonetheless, we strongly oppose any proposal against *H. pylori* eradication in infected patients, given its classification as a Group 1 carcinogen by the International Agency for Research on Cancer (IARC), indicating its potential carcinogenicity for gastric mucosa.

Overall, these preliminary association studies emphasize the necessity for broader, prospective, and more standardized research on this subject, potentially paving the way for new insights into the environmental aethiology of IBDs.

In conclusion, the relationship between *H. pylori* infection and IBDs and its potential protective or detrimental role in IBD pathogenesis, remains a topic of debate, with conflicting evidence from different studies. Whether this association is coincidental, epiphenomenal, or mechanistic remains unclear. Discrepancies among studies can be attributed to various confounding factors, including differences in study power, study periods, geographic variations, and methods used for *H. pylori* detection. The true impact of *H. pylori* presence, particularly its apparent protective effect, needs clarification through additional studies. Indeed, such effects could help in the development of new therapeutic strategies against IBD, which remains an important clinical challenge. Although evidence shows a tendency to view *H. pylori* as a protective factor, the classification of this bacterium as a Group 1 carcinogen according to IARC underscores the need for eradication in every clinical setting. Given the critical nature of the topic, it is in any case mandated that we need to have further large-scale studies to evaluate the relationship between *H. pylori* and IBDs, testing for the presence of the bacterium at each new diagnosis of the disease, so as to have more details on the correlation with time interval of IBD development and its clinical features.

## Data Availability

Not applicable.
